# Modelling of Chaotic Processes with Caputo Fractional Order Derivative

**DOI:** 10.3390/e22091027

**Published:** 2020-09-14

**Authors:** Kolade M. Owolabi, José Francisco Gómez-Aguilar, G. Fernández-Anaya, J. E. Lavín-Delgado, E. Hernández-Castillo

**Affiliations:** 1 Faculty of Mathematics and Statistics, Ton Duc Thang University, Ho Chi Minh City 700000, Vietnam; koladematthewowoabi@tdtu.edu.vn; 2 Department of Mathematical Sciences, Federal University of Technology, Akure PMB 704, Ondo State, Nigeria; 3 CONACyT-Tecnológico Nacional de México/CENIDET, Interior Internado Palmira S/N, Col. Palmira, Cuernavaca C.P. 62490, Morelos, Mexico; 4 Departamento de Física y Matemáticas, Universidad Iberoamericana, CDMX Mexico C.P. 01219, Mexico; guillermo.fernandez@ibero.mx; 5 Tecnológico Nacional de México/CENIDET, Interior Internado Palmira S/N, Col. Palmira, Cuernavaca C.P. 62490, Morelos, Mexico; jorge.lavin@cenidet.edu.mx; 6 Instituto Tecnológico de Tlalnepantla, Av. Instituto Tecnológico S/N, Col. La Comunidad, Tlalnepantla de Baz C.P. 54070, Mexico; eric.hc@tlalnepantla.tecnm.mx

**Keywords:** chaotic dynamics, chebyshev spectral method, fractional differential equation, spatiotemporal oscillations, stability analysis

## Abstract

Chaotic dynamical systems are studied in this paper. In the models, integer order time derivatives are replaced with the Caputo fractional order counterparts. A Chebyshev spectral method is presented for the numerical approximation. In each of the systems considered, linear stability analysis is established. A range of chaotic behaviours are obtained at the instances of fractional power which show the evolution of the species in time and space.

## 1. Introduction

Fractional differential systems are largely encountered in various fields of applied sciences and engineering [1,2,3,4,5]. The study of differential equations of noninteger-order derivative have made tremendous development in terms of theory and application. It encompasses the general extension of classical order integrals and derivatives to fractional order counterparts.

The concept of fractional derivative has greatly been extended to various fractional ordinary differential equations, for instance the fractional Sturm–Liouville, Cauchy, and Gauss equations [1], with lots of applications in modelling of partial differential equations such as the complex cubic-quintic Ginzburg–Landau equation, Gray–Scott system [6], Burgers equation, Fisher equation, Fokker–Planck equation [7], and a range of fractional advection-reaction-diffusion problems, see [1,3,8] and references given therein. The main advantage of such models is the introduction of a fractional parameter, say γ which can be used to model non-Markovian behaviour of spatial or temporal processes. This technique has emerged over the years as generalisations of many classic scenarios in mathematical physics.

Over the years, it is understood that many phenomena in application areas of science can be analysed successfully by models with the aid of mathematical tools from fractional calculus. A lot of researchers have realised the importance of fractional differential equations in describing real-life phenomena. A large number of fractional models have been suggested and examined in different areas such as the merits of non-integer order equations over the integer-order cases, analytical results, numerical techniques, and a host of others.

Fractional differential equations have been studied by many researches with different methods of solutions [1,2,9]. Many authors have also investigated analytic results based on the existence and uniqueness of solutions to fractional differential equations [1,3,10]. It should be mentioned that most fractional differential models encountered in science and engineering do have exact solutions and as a result, a viable and approximate numerical technique is required. So, to find efficient and accurate numerical methods to handle this class of problems has been a challenge over the years. Among several analytic and numerical methods that have been proposed includes the Adomian decomposition method, finite difference technique, the homotopy-perturbation approach, predictor-corrector method, collocation spectral method, Fourier spectral method, among several others [11,12,13].

The aim of the present paper is to approximate a chaotic process using the Chebyshev spectral method. Various chaotic processes have been modelled by a number of fractional derivatives. Chaotic differential equations are largely encountered in various fields of engineering, physics, chemistry, economics, and other related applied science subjects [14,15,16]. The main advantage of spectral methods relies mainly in their accuracy for a given number of unknowns. For smooth problems especially in geometries, spectral methods offer exponential rates of convergence with good spectral accuracy [6,11,17,18].

The remainder part of this paper is structured as follows. Some mathematical preliminaries based on basic definitions of fractional calculus and some important properties of Chebyshev spectral polynomials are introduced in Section 2. The derivation of an approximate formula for a chaotic system is given in Section 3. Some numerical experiment to reveal the behaviour of such a system is reported in Section 4. The paper is concluded with the last section.

## 2. Useful Preliminaries

Some useful definitions, results, and properties of fractional calculus, and Chebyshev polynomials are reported in this section.

Given a general time-fractional differential equation:

(1) $$\begin{array}{ccc} D^{\gamma }g\left(t\right) & = & f(t,D^{\beta }g\left(t\right)),\;\;\mathrm{for}\;1\leq t>0,\\ g^{\left(s\right)}\left(0\right) & = & \sigma_{s},\;\;s=0,1,2,\ldots ,n-1 \end{array}$$

where n−1<γ<n and m−1<β<m, for m,n∈N,n−1≥m, $D^{\gamma }$ denotes the Caputo fractional derivative operator of order γ, and f∈C([0,1]×R). For the existence of a solution, the nonlinear function *f* is required to satisfy the condition: ∃ two functions, say a,b∈([0,1],[0,∞)):∀1≥t≥0 and for each w∈R, we have |f(t,w)|≤a(t)|w|+b(t). Similarly for the uniqueness result, the term *f* must satisfy the condition: ∃a,b∈([0,1],[0,∞)):∀1≥t>0 and any w,z∈R, we have |f(t,w)−f(t,z)|≤a(t)|w−z|.

The Caputo fractional derivative of order γ>0 is defined as:

(2) $${}^{C}D^{\gamma }g\left(x\right)=\frac{1}{\Gamma (n-\gamma )}\int_{0}^{x}\frac{g^{\left(n\right)}\left(\tau \right)}{{\left(x-\tau \right)}^{\gamma -n+1}}d\tau ,$$

where x>0 and n−1<γ≤n for n∈N and Γ(x) is the Gamma function.

The Caputo derivative of a constant is defined as:

(3) $${}^{C}D^{\gamma }A=0,\;\;\mathrm{with}\;A,\;a\;\mathrm{constant}.$$

Also,

(4) $${}^{C}D^{\gamma }x^{\alpha }=\left\{\begin{array}{cc} 0 & \mathrm{for}\;\alpha \in N_{0}\;\mathrm{and}\;\alpha <\lceil \gamma \rceil ,\\ \frac{\Gamma (\alpha +1)}{\Gamma (\alpha +1-\gamma )}x^{\alpha -\gamma } & \mathrm{for}\;\alpha \in N_{0}\;\mathrm{and}\;\alpha \leq \lceil \gamma \rceil . \end{array}\right.$$

**Theorem** **1** ([10])**.** *Let m−1<β<γ<m for m∈N. Assume that the following hypothesis holds: ($h_{1}$) g:[0,1]×R→R is a continuously differentiable function, ($h_{2}$) g(0,0)=0 and g(t,0)≠0 on a compact subinterval of (0,] and ($h_{3}$) there exist ϵ>1, 1>ρ>0 and f(t)∈C([0,1],[0,∞]) such that:*

$$\frac{1}{\Gamma (\gamma -\beta )}\underset{0\leq t\leq 1}{\sup}\int_{0}^{t}{\left(t-\tau \right)}^{\gamma -\beta -1}f\left(\tau \right)d\tau \leq 1-\rho .$$

$$0<R:=\frac{1}{\Gamma (\gamma -\beta )}\underset{0\leq t\leq 1}{\sup}\int_{0}^{t}{\left(t-\tau \right)}^{\gamma -\beta -1}\left|g\left(\tau ,0\right)\right|d\tau <\infty$$

*and for every a,b∈C([0,∞]) with $0\leq \left|a\left(t\right)|,|b\left(t\right)\right|\leq \frac{1}{\rho }R$ for 1≥t≥0, then:*

|g(t,a(t))−g(t,b(t))|≤f(t)|a(t)−b(t)|for1≥t≥0.

*Then Equation (1) has a unique solution.*

For details of a similar proof of Theorem 1, see [10,19].

The Chebyshev polynomial on interval [−1,1] is determined using the recurrence formula [12,20]:

$$G_{n+1}\left(x\right)=2xG_{n}\left(x\right)-G_{n-1}\left(x\right),G_{0}\left(x\right)=1,G_{1}\left(x\right)=x,\mathrm{for}\;n=1,2,\ldots$$

The analytic form of the Chebyshev polynomials $G_{n}\left(x\right)$ is defined by:

(5) $$G_{n}\left(x\right)=n\sum_{k=0}^{\left\lfloor \frac{n}{2}\right\rfloor }{\left(-1\right)}^{k}2^{n-2k-1}\frac{(n-k-1)!}{k!(n-2k)!}x^{n-2k},\;\;n=2,3,\ldots ,$$

where $\left\lfloor \frac{n}{2}\right\rfloor$ stands for the integer case of n/2. The orthogonality property is given:

(6) $$\int_{-1}^{1}\frac{G_{k}\left(x\right)G_{s}\left(x\right)}{\sqrt{1-x^{2}}}dx=\left\{\begin{array}{cc} \pi & \mathrm{for}\;k=s=0,\\ \frac{\pi }{2} & \mathrm{for}\;k=s\neq 0,\\ 0 & \mathrm{for}\;k\neq s, \end{array}\right.$$

It is necessary to introduce a change of variable x=2t−1 to enable us to apply the Chebyshev polynomial on interval [0,1]. Hence, we follow [12] and define the shifted Chebyshev polynomial as:

$$G^{*}\left(t\right)=G_{n}\left(2t-1\right)=G_{2n}\left(\sqrt{t}\right),$$

and its analytic form is given by:

(7) $$G_{n}^{*}\left(t\right)=n\sum_{r=0}^{n}{\left(-1\right)}^{n-r}\frac{(n+r-1)!}{(2r)!(n-r)!}t^{r},\;\;n=2,3,\ldots$$

## 3. Fractional-Order Chaotic Systems and Formulation of Approximate Scheme

In this section, we introduce a three- and four-scroll chaotic attractor systems [21,22] and examine their linear stability analysis. Later, we derive an approximate formula based on the Chebyshev spectral method for their numerical approximations.

### 3.1. Chaotic System

Two important chaotic systems which exist in classical order forms, which have been studied and applied in various areas of finance, stock exchange, cryptology, population dynamics, etc. are reformulated here in the sense of the Caputo fractional order models. Each system is also examined for linear stability analysis.

#### 3.1.1. Three Dimensional Fractional Chaotic System

The fractional order chaotic system of three components is described by the following differential equations [23]:

(8) $$\begin{array}{ccc} {}^{C}D_{t}^{\gamma }u\left(t\right) & = & f_{1}\left(u,v,w\right)=a_{1}\left(u\left(t\right)-v\left(t\right)\right),\\ {}^{C}D_{t}^{\gamma }v\left(t\right) & = & f_{2}\left(u,v,w\right)=-4a_{1}v\left(t\right)+u\left(t\right)w\left(t\right)+a_{2}u^{3}\left(t\right),\\ {}^{C}D_{t}^{\gamma }w\left(t\right) & = & f_{3}\left(u,v,w\right)=-a_{1}a_{4}w\left(t\right)+u^{3}\left(t\right)v\left(t\right)+a_{3}w^{2}\left(t\right), \end{array}$$

where u(t),v(t) and w(t) denote state variables and $a_{i}>0$ for i=1(1)4 are parameters.

To examine system (8) for equilibrium points, we set ${}^{C}D_{t}^{\gamma }=f_{i}\left(u,v,w\right)=0,i=1,2,3$. That is,

(9) $$\begin{array}{ccc} 0 & = & a_{1}\left(u\left(t\right)-v\left(t\right)\right),\\ 0 & = & -4a_{1}v\left(t\right)+u\left(t\right)w\left(t\right)+a_{2}u^{3}\left(t\right),\\ 0 & = & -a_{1}a_{4}w\left(t\right)+u^{3}\left(t\right)v\left(t\right)+a_{3}w^{2}\left(t\right). \end{array}$$

With this development, it is obvious that system (8) has four steady states, which correspond to $E_{0}=\left(0,0,0\right)$ which is the washout state, $\bar{E}=\left(0,0,\frac{a_{1}a_{4}}{a_{3}}\right)$, this point corresponds to the existence of state variable *w* only. The other two equilibrium points are given by:

$$E_{+}^{*}=\left(\sqrt{\frac{4a_{1}-w^{*}}{a_{2}}},\sqrt{\frac{4a_{1}-w^{*}}{a_{2}}},\frac{a_{1}\left(a_{4}a_{2}^{2}+8\right)+a_{1}a_{2}\sqrt{a_{4}^{2}a_{2}2+16a_{4}-64a_{3}}}{2a_{3}a_{2}^{2}+2}\right)$$

and

$$E_{-}^{*}=\left(-\sqrt{\frac{4a_{1}-w^{*}}{a_{2}}},-\sqrt{\frac{4a_{1}-w^{*}}{a_{2}}},\frac{a_{1}\left(a_{4}a_{2}^{2}+8\right)+a_{1}a_{2}\sqrt{a_{4}^{2}a_{2}2+16a_{4}-64a_{3}}}{2a_{3}a_{2}^{2}+2}\right)$$

where $w^{*}=\frac{a_{1}\left(a_{4}a_{2}^{2}+8\right)+a_{1}a_{2}\sqrt{a_{4}^{2}a_{2}2+16a_{4}-64a_{3}}}{2(a_{3}a_{2}^{2}+1)}$. The Jacobian or community matrix of system (8) corresponds to:

(10) $$A_{u,v,w}=\left(\begin{array}{ccc} a_{1} & -a_{1} & 0\\ 3a_{2}u^{2}+w & -4a_{1} & u\\ 3u^{2}v & u^{3} & 2a_{3}w-a_{1}a_{4} \end{array}\right).$$

Let det(λI−A(0,0,0))=0, we obtain the characteristic equation:

$$\lambda^{3}+k_{1}\lambda^{2}+k_{2}\lambda -k_{3},$$

where $k_{i},i=1,2,3=\left(1.8,1.61,35\right)$, respectively. Clearly on solving further, we obtain the eigenvalues as $\lambda_{1}=toe1.80,\lambda_{2}=-7.20$, and $\lambda_{3}=-7.20$. Since one of the roots of the characteristic equation above is a positive real number, the remaining two are real-negative, then point $E^{0}$ is saddle and unstable. Following a similar procedure, the corresponding eigenvalues at point $\bar{E}$ as $\lambda_{1}=6.77$, $\lambda_{2}=-12.17$ and $\lambda_{1}=2.70$, therefore since one of the three eigenvalues has an opposite sign, the point $\bar{E}$ is also unstable.

Next, by considering the interior point $E_{\pm }^{*}$, we have eigenvalues $\left(\lambda_{1},\lambda_{2},\lambda_{3}\right)=\left(0.83-3.83j,0.83+3.83j,-10.75\right)$ and $\left(\lambda_{1},\lambda_{2},\lambda_{3}\right)=\left(0.83-3.83j,0.83+3.83j,-10.75\right)$, respectively. In both equilibrium points $E_{+}^{*}$ and $E_{-}^{*}$, we have $\lambda_{3}$ as a negative real number, whereas $\lambda_{1}$ and $\lambda_{2}$ are a conjugate pair of complex eigenvalues with positive real parts. This indicates that the nontrivial points $E_{+}^{*}$ and $E_{-}^{*}$ are saddle-focus. Hence, these steady states are unstable points.

**Figure 1:**
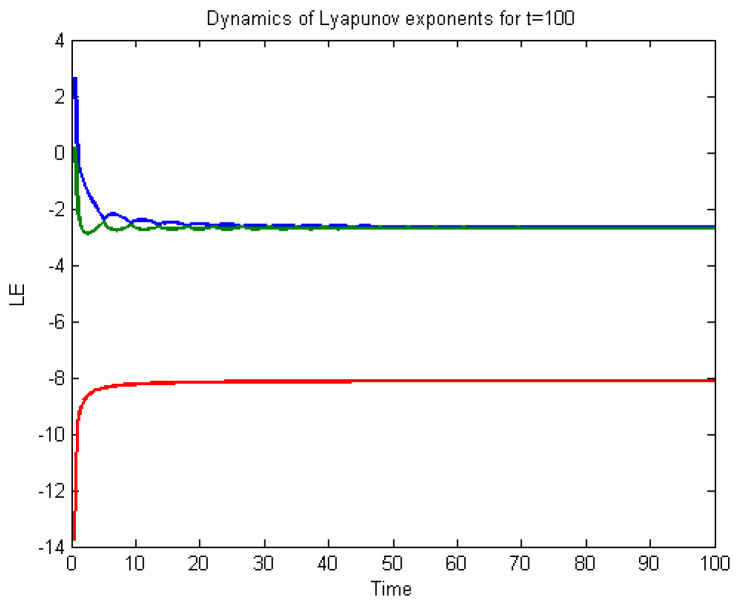
Dynamic of the Lyapunov exponent for system (8).

**Figure 2:**
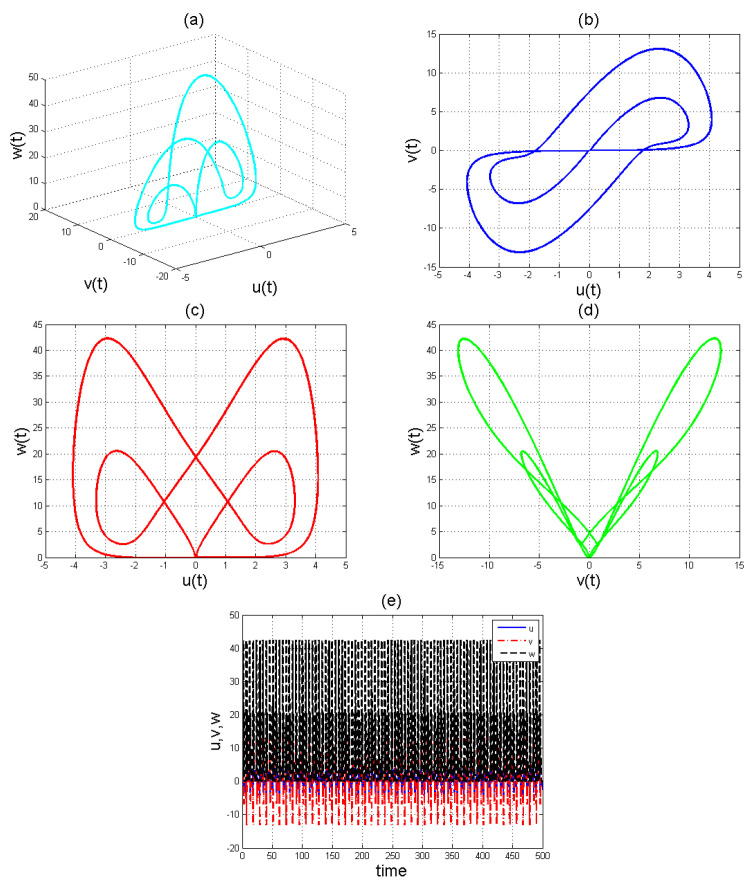
Chaotic evolution of fractional system (8) at γ=0.38. Plots (**a**) 3D chaotic attractor, (**b**–**d**) are 2D chaotic distributions, and (**e**) chaotic time-series solution.

**Figure 3:**
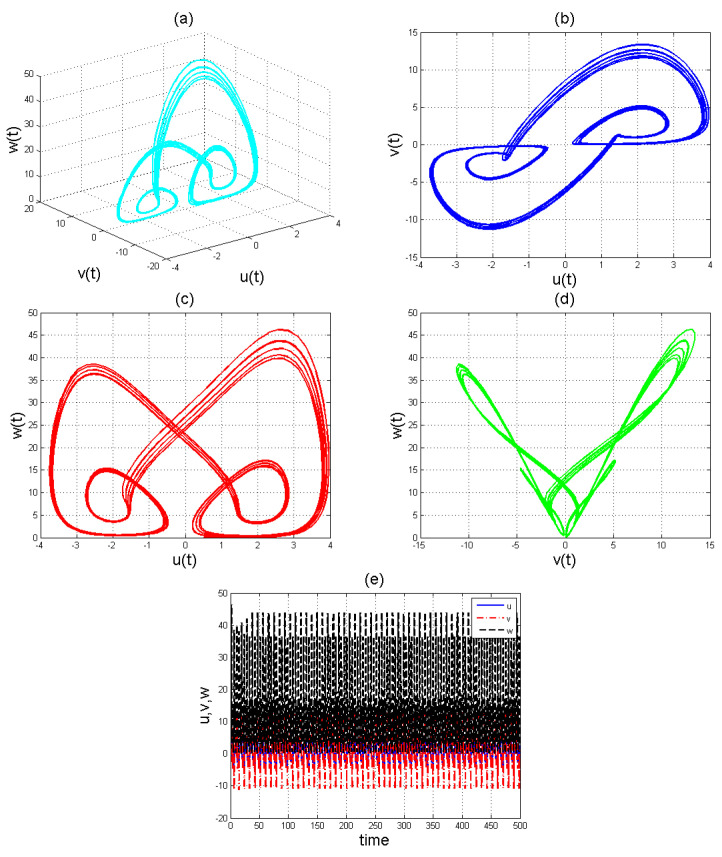
Chaotic evolution of fractional system (8) at γ=0.51. Plots (**a**) 3D chaotic attractor, (**b**–**d**) are 2D chaotic distributions, and (**e**) chaotic time-series solution.

**Figure 4:**
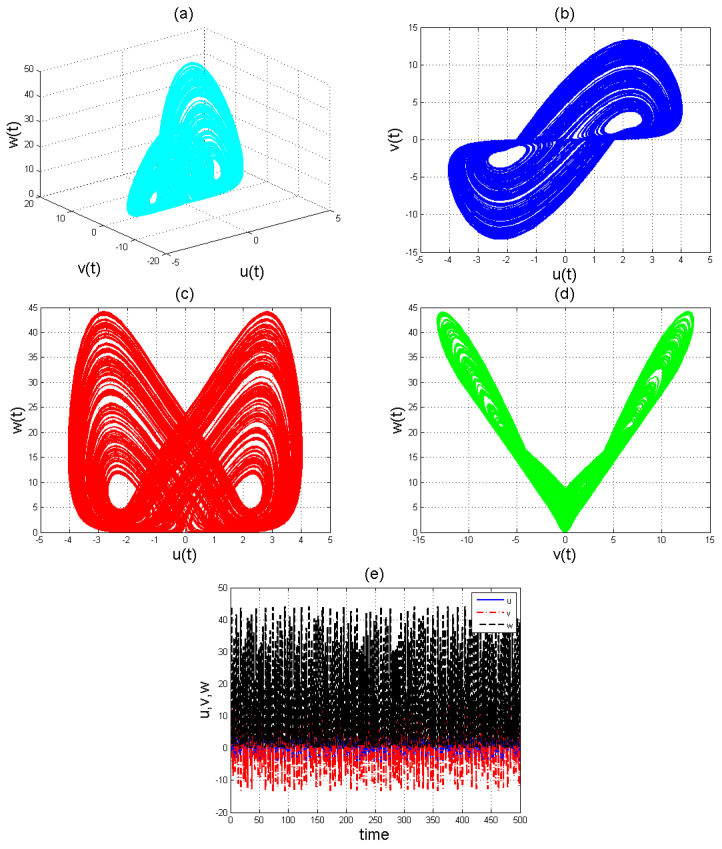
Chaotic evolution of fractional system (8) at γ=0.99. Plots (**a**) 3D chaotic attractor, (**b**–**d**) are 2D chaotic distributions, and (**e**) chaotic time-series solution.

To analyse the nonlinear behaviour of the dynamic system (8), we consider its Lyapunov exponent, which measures the exponential rates of divergence or convergence of trajectories. It should be mentioned that if at least one of the Lyapunov exponent (LE) is positive, then we have a chaotic system. With parameters $a_{1}=1.8,a_{2}=0.12,a_{3}=-0.07;a_{4}=1.5;$ and $a_{4}=1.5$ with $\left(u_{0},v_{0},w_{0}\right)=\left(2.6,1.8,2.5\right)$, we get the corresponding as $LE^{1}=-2.679038,LE^{2}=-2.692823$, and $LE^{3}=-8.113770,t=100.0000-2.679038-2.692823-8.113770$. Please see Figure 1) for the dynamic of Lyapunov exponents computed at t=100. Figure 2, Figure 3 and Figure 4 shows the chaotic evolution of fractional system (8) for γ=0.38, γ=0.51 and γ=0.99, arbitrarily chosen.

#### 3.1.2. Four-Scroll Fractional-Order Chaotic System

A four-scroll Caputo fractional order chaotic system with nine parameters is given as [21,22]:

(11) $$\begin{array}{ccc} {}^{C}D_{t}^{\gamma }u\left(t\right) & = & f_{1}\left(u,v,w\right)=b_{1}v\left(t\right)-b_{2}u\left(t\right)+b_{3}u\left(t\right)w\left(t\right),\\ {}^{C}D_{t}^{\gamma }v\left(t\right) & = & f_{2}\left(u,v,w\right)=-b_{4}u\left(t\right)w\left(t\right)-b_{5}u\left(t\right)+b_{6}v\left(t\right)w\left(t\right)+b_{7}u\left(t\right),\\ {}^{C}D_{t}^{\gamma }w\left(t\right) & = & f_{3}\left(u,v,w\right)=b_{8}-b_{9}v^{2}\left(t\right), \end{array}$$

with ${}^{C}D_{t}^{\gamma }=f_{i}\left(u,v,w\right)=0$, the Caputo chaotic system (11) have equilibrium states:

$$\left(u,v,w\right)=E_{\pm }=\left({\bar{u}}_{\pm },{\bar{v}}_{\pm },{\bar{w}}_{\pm }\right)$$

where,

$${\bar{u}}_{+}=\frac{b_{1}{\bar{v}}_{+}}{b_{2}-b3{\bar{w}}_{+}},\;\;{\bar{u}}_{-}=\frac{b_{1}{\bar{v}}_{-}}{b_{2}-b3{\bar{w}}_{-}},$$

$${\bar{v}}_{+}=\sqrt{\frac{b_{8}}{b_{9}}},\;\;{\bar{v}}_{-}=-\sqrt{\frac{b_{8}}{b_{9}}}$$

and

$${\bar{w}}_{+}=\frac{b_{2}b_{6}-b_{1}b_{4}}{2b_{3}b_{6}}+\frac{\sqrt{{\left(b_{2}b_{6}-b_{1}b_{4}\right)}^{2}-4b_{1}b_{3}b_{6}\left(b_{5}-b_{7}\right)}}{2b_{3}b_{6}},$$

$${\bar{w}}_{-}=\frac{b_{2}b_{6}-b_{1}b_{4}}{2b_{3}b_{6}}-\frac{\sqrt{{\left(b_{2}b_{6}-b_{1}b_{4}\right)}^{2}-4b_{1}b_{3}b_{6}\left(b_{5}-b_{7}\right)}}{2b_{3}b_{6}}.$$

The linear stability is obtained by solving the characteristic equation:

(12) $$\lambda^{3}+\xi_{2}\lambda^{2}+\xi_{1}\lambda +\xi_{0}=0,$$

where,

$$\xi_{2}=b_{2}-\bar{w}\left(b_{3}+b_{6}\right),$$

$$\xi_{1}=\frac{2b_{9}\bar{u}\bar{v}\left(b_{5}-b_{7}\right)}{\bar{w}},$$

and

$$\xi_{0}=\frac{2b_{8}\left[b_{1}\left(b_{5}-b_{7}\right)-b_{3}b_{6}{\bar{w}}^{2}\right]}{\bar{w}}.$$

**Figure 5:**
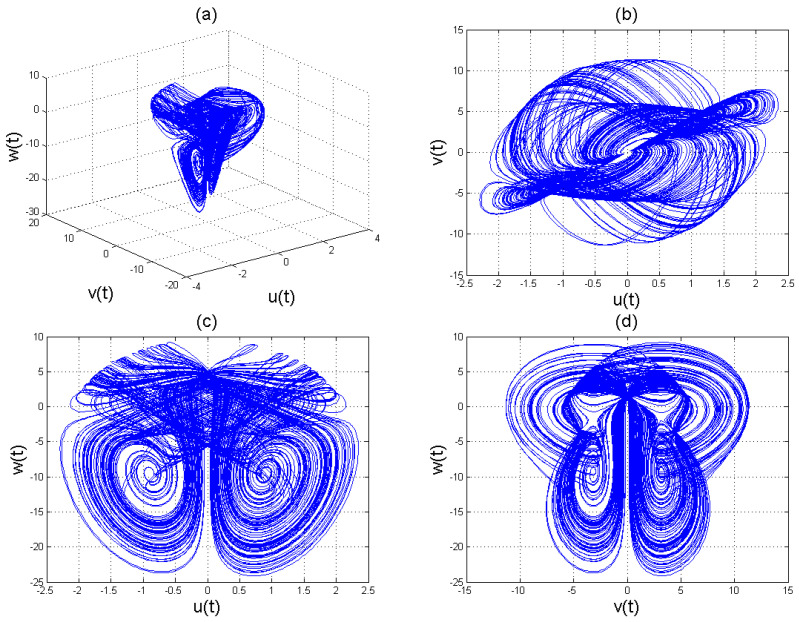
Chaotic attractors involving the Caputo fractional order derivative for the four-scroll chaotic system (11) with γ=1.

**Figure 6:**
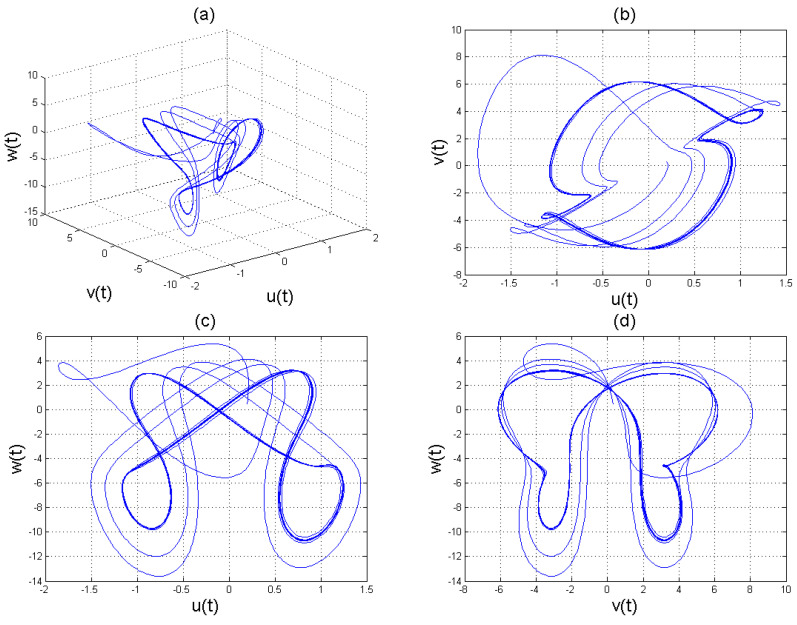
Chaotic attractors involving the Caputo fractional order derivative for the four-scroll chaotic system (11) with γ=0.38.

**Figure 7:**
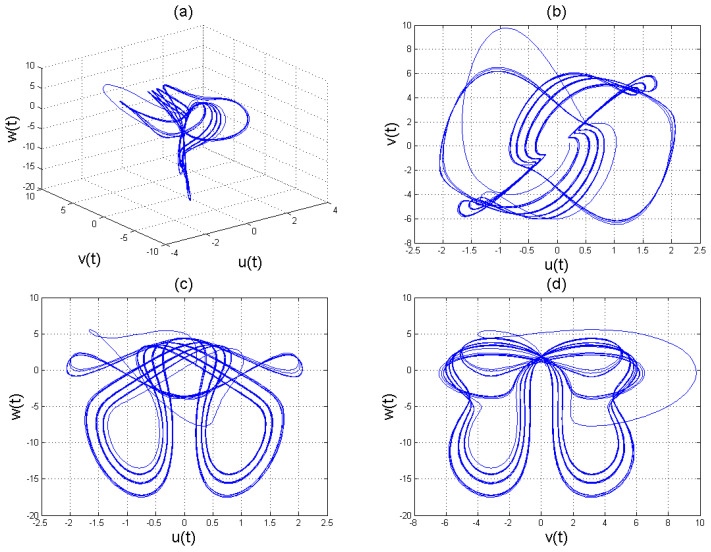
Chaotic attractors involving the Caputo fractional order derivative for the four-scroll chaotic system (11) with γ=0.50.

**Figure 8:**
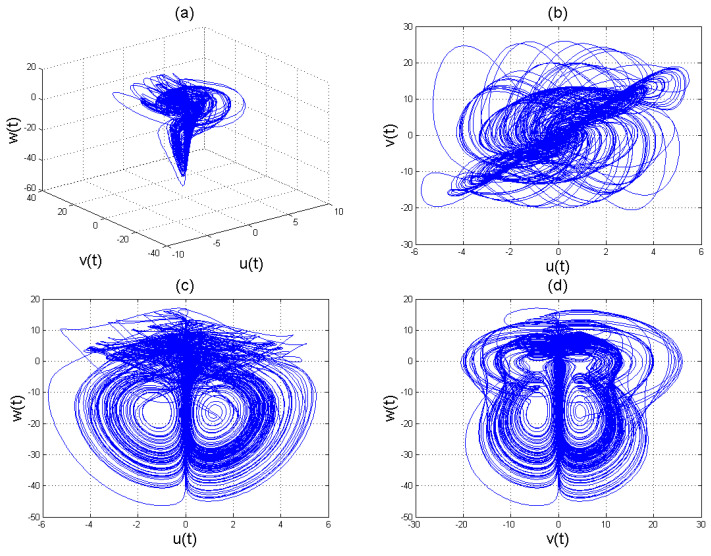
Chaotic attractors involving the Caputo fractional order derivative for the four-scroll chaotic system (11) with γ=0.61.

**Figure 9:**
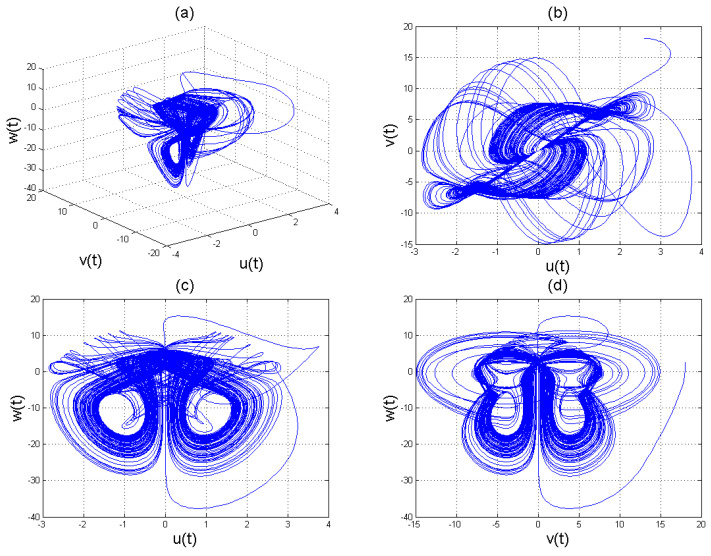
Chaotic attractors involving the Caputo fractional order derivative for the four-scroll chaotic system (11) with γ=0.84.

**Figure 10:**
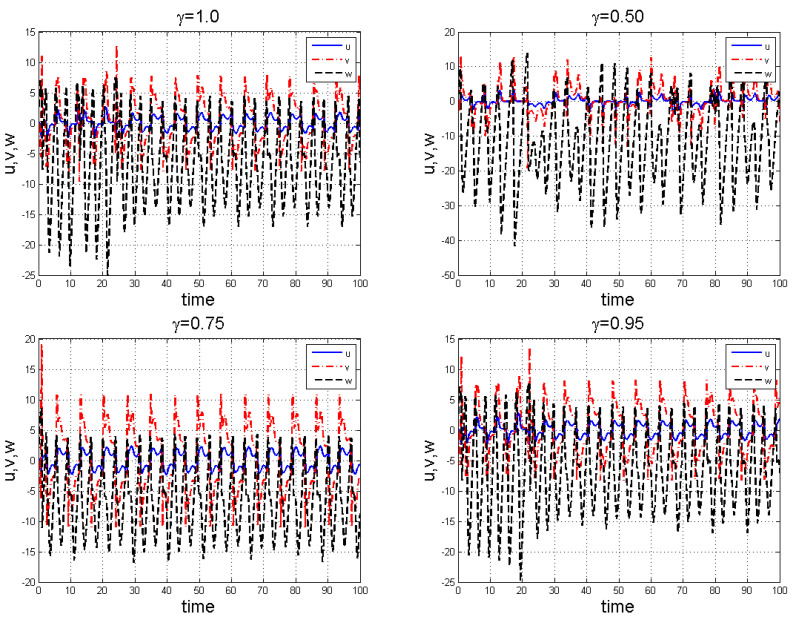
Time series showing chaotic evolution of system (11) for several values of γ.

With $\varpi ={\left(b_{2}b_{6}-b_{1}b_{4}\right)}^{2}-4b_{1}b_{3}b_{6}\left(b_{5}-b_{7}\right)=0$, we have saddle-node bifurcation, when ϖ>0 we have two fixed points. One of the fixed points is stable while the other unstable for ${\bar{w}}_{\pm }$. Figure 5, Figure 6, Figure 7, Figure 8 and Figure 9 for γ=1, γ=0.38, γ=0.50, γ=0.61, and γ=0.84, arbitrarily chosen. Also Figure 10 shows the time series chaotic evolution of system (11) for several values of γ.

### 3.2. Formulation of Approximate Method

The function g(t) in space [0,1] can be defined in terms of shifted Chebyshev polynomials as:

(13) $$g\left(t\right)=\sum_{k=0}^{\infty }\omega_{k}G_{k}^{*}\left(t\right),$$

with $\omega_{k}$ as coefficients, expressed in the form [20]:

(14) $$\omega_{k}=\frac{2}{\pi \hbar_{k}}\int_{0}^{1}\frac{g\left(t\right)G_{k}^{*}\left(t\right)}{\sqrt{t-t^{2}}}dt,\;\;\hbar_{0}=2,\;\;\hbar_{k}=1,\;k=1,2,\ldots .$$

It is customary to consider only the first (x+1) terms of the shifted Chebyshev polynomials. In such that:

(15) $$g_{x}\left(t\right)=\sum_{k=0}^{x}\omega_{k}G_{k}^{*}\left(t\right).$$

The approximate formula for fractional derivative of order γ>0 of $g_{x}\left(t\right)$ using the above Chebyshev polynomials, to have:

(16) $$D_{t}^{\gamma }\left(g_{x}\left(t\right)\right)=\sum_{k=\lceil \gamma \rceil }^{x}\sum_{r=\lceil \gamma \rceil }^{k}\omega_{k}f_{k,r}^{\left(\gamma \right)}t^{r-\gamma },$$

where,

$$f_{k,r}^{\left(\gamma \right)}={\left(-1\right)}^{k-r}\frac{2^{2r}k\left(k+r-1\right)!\Gamma \left(r+1\right)}{\left(k-r)!(2r)!\Gamma (r+1-\gamma \right)}.$$

So, the Caputo fractional operator of order γ>0 for the shifted Chebyshev polynomials is given in the form:

(17) $${}^{C}D_{0}^{\gamma }\left[G_{k}^{*}\left(t\right)\right]=\sum_{r=\lceil \gamma \rceil }^{k}\sum_{s=0}^{r-\lceil \gamma \rceil }\chi_{k,r,s}G_{s}^{*}\left(t\right),$$

where,

$$\chi_{k,r,s}=\frac{{\left(-1\right)}^{k-r}2k\left(k+r-1\right)!\Gamma \left(r-\gamma +\frac{1}{2}\right)}{\hbar_{s}\Gamma \left(r+\frac{1}{2}\right)\left(k-r\right)!\Gamma \left(r-\gamma -s+1\right)\Gamma \left(r+s-\gamma +1\right)},\;\mathrm{for}\;s=0,1,2,\ldots$$

The error, denoted as $|E_{r}\left(x\right)|$ is calculated as $|D^{\gamma }g\left(t\right)-D^{\gamma }g_{x}\left(t\right)|$ in approximating $D^{\gamma }g\left(t\right)$ by $D^{\gamma }g_{x}\left(t\right)$ which is bounded by:

$$|E_{r}\left(x\right)|\leq \left|\sum_{k=x+1}^{\infty }\omega_{k}\left(\sum_{r=\lceil \gamma \rceil }^{k}\sum_{s=0}^{r-\lceil \gamma \rceil }\chi_{k,r,s}\right)\right|.$$

For details, readers are referred to [20].

Next, we provide a solution procedure of the Caputo fractional chaotic system by first approximating u(t),v(t) and w(t) as:

(18) $$\begin{array}{c} u_{n}\left(t\right)=\sum_{k=0}^{n}x_{k}G_{k}^{*}\left(t\right),\;\;\;v_{n}\left(t\right)=\sum_{k=0}^{n}y_{k}G_{k}^{*}\left(t\right),\;\;\;w_{n}\left(t\right)=\sum_{k=0}^{n}z_{k}G_{k}^{*}\left(t\right). \end{array}$$

From Equations (8) and (16) we obtain:

(19) $$\begin{array}{ccc} & & \sum_{k=\lceil \gamma \rceil }^{n}\sum_{r=\lceil \gamma \rceil }^{k}x_{k}f_{i,r}^{\left(\gamma \right)}t^{r-\gamma }=a_{1}\left(\sum_{k=0}^{n}x_{k}G_{k}^{*}\left(t\right)-\sum_{k=0}^{n}y_{k}G_{k}^{*}\left(t\right)\right),\\ & & \sum_{k=\lceil \gamma \rceil }^{n}\sum_{r=\lceil \gamma \rceil }^{k}y_{k}f_{i,r}^{\left(\gamma \right)}t^{r-\gamma }=-4a_{1}\sum_{k=0}^{n}y_{k}G_{k}^{*}\left(t\right)+\left(\sum_{k=0}^{n}x_{k}G_{k}^{*}\left(t\right)\right)\left(\sum_{k=0}^{n}z_{k}G_{k}^{*}\left(t\right)\right)\\ & & \;+a_{2}{\left(\sum_{k=0}^{n}z_{k}G_{k}^{*}\left(t\right)\right)}^{3}\\ & & \sum_{k=\lceil \gamma \rceil }^{n}\sum_{r=\lceil \gamma \rceil }^{k}z_{k}f_{i,r}^{\left(\gamma \right)}t^{r-\gamma }=-a_{1}a_{4}\sum_{k=0}^{n}z_{k}G_{k}^{*}\left(t\right)+{\left(\sum_{k=0}^{n}x_{k}G_{k}^{*}\left(t\right)\right)}^{3}\left(\sum_{k=0}^{n}y_{k}G_{k}^{*}\left(t\right)\right)\\ & & \;+a_{3}{\left(\sum_{k=0}^{n}z_{k}G_{k}^{*}\left(t\right)\right)}^{2}. \end{array}$$

Next, we collocate (19) at (n+1−⌈γ⌉) points $t_{\eta }\left(\eta =0,1,2,\ldots ,m+1-\left\lceil \gamma \right\rceil \right)$ to get:

(20) $$\begin{array}{ccc} & & \sum_{k=\lceil \gamma \rceil }^{n}\sum_{r=\lceil \gamma \rceil }^{k}x_{k}f_{i,r}^{\left(\gamma \right)}t_{\eta }^{r-\gamma }=a_{1}\left(\sum_{k=0}^{n}x_{k}G_{k}^{*}\left(t_{\eta }\right)-\sum_{k=0}^{n}y_{k}G_{k}^{*}\left(t_{\eta }\right)\right),\\ & & \sum_{k=\lceil \gamma \rceil }^{n}\sum_{r=\lceil \gamma \rceil }^{k}y_{k}f_{i,r}^{\left(\gamma \right)}t_{\eta }^{r-\gamma }=-4a_{1}\sum_{k=0}^{n}y_{k}G_{k}^{*}\left(t_{\eta }\right)+\left(\sum_{k=0}^{n}x_{k}G_{k}^{*}\left(t_{\eta }\right)\right)\left(\sum_{k=0}^{n}z_{k}G_{k}^{*}\left(t_{\eta }\right)\right)\\ & & \;+a_{2}{\left(\sum_{k=0}^{n}z_{k}G_{k}^{*}\left(t_{\eta }\right)\right)}^{3}\\ & & \sum_{k=\lceil \gamma \rceil }^{n}\sum_{r=\lceil \gamma \rceil }^{k}z_{k}f_{i,r}^{\left(\gamma \right)}t_{\eta }^{r-\gamma }=-a_{1}a_{4}\sum_{k=0}^{n}z_{k}G_{k}^{*}\left(t_{\eta }\right)+{\left(\sum_{k=0}^{n}x_{k}G_{k}^{*}\left(t_{\eta }\right)\right)}^{3}\left(\sum_{k=0}^{n}y_{k}G_{k}^{*}\left(t_{\eta }\right)\right)\\ & & \;+a_{3}{\left(\sum_{k=0}^{n}z_{k}G_{k}^{*}\left(t_{\eta }\right)\right)}^{2}. \end{array}$$

We use the roots of shifted Chebyshev polynomials $G_{n+1-\lceil \gamma \rceil }^{*}\left(t\right)$ to get suitable collocation points. By putting (18) into initial conditions of the form:

$$u\left(0\right)=u_{0},\;v\left(0\right)=v_{0},\;\mathrm{and}\;w\left(0\right)=w_{0}$$

we obtain:

(21) $$\sum_{k=0}^{n}{\left(-1\right)}^{k}x_{k}=u_{0},\;\;\sum_{k=0}^{n}{\left(-1\right)}^{k}y_{k}=v_{0},\;\;\sum_{k=0}^{n}{\left(-1\right)}^{k}z_{k}=w_{0}.$$

So Equations (20) and (21) result to a system on nonlinear algebraic equations which can be solved by any iterative method. In the present case, we implement with inbuilt Ode45 in Matlab.

## 4. Experimental Results

Under this segment, we briefly report the behaviour of fractional order chaotic systems (8) and (11) governed by the influence of fractional index γ in the sense of the Caputo operator.

In the simulation framework for system (8), we simulate with initial conditions $\left(u_{0},v_{0},w_{0}\right)=\left(2.6,1.8,2.5\right)$ and parameter values $a_{1}=1.8$, $a_{2}=0.12$, $a_{3}=-0.07$, and $a_{4}=1.5$ to obtain chaotic patterns as displayed in Figure 2, Figure 3 and Figure 4 at different instances of γ. It was observed that, regardless of the value of γ chosen, system (8) exhibits a chaotic pattern.

For the four-scroll fractional chaotic system (11), with parameter choice:

(22) $$\begin{array}{c} b_{1}=1,b_{2}=0.7,b_{3}=0.3,b_{4}=4,b_{5}=4.4,b_{6}=1,b_{7}=0.1,b_{8}=10,b_{9}=1, \end{array}$$

we observe the chaotic results as shown in Figure 5, Figure 6, Figure 7, Figure 8, Figure 9 and Figure 10 for various γ. It is clear that the chaotic pattern formation process for integer and fractional-order cases are almost similar.

**Figure 11:**
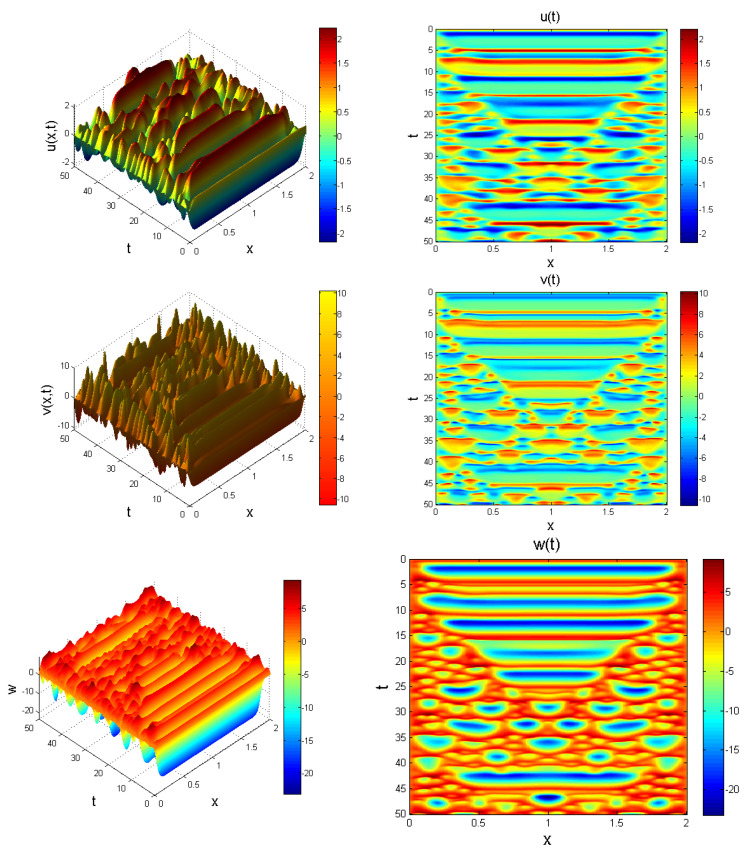
Evolution of system (23) at γ=0.50, x∈[0,2] for t=50 Chaotic behaviour is noticeable.

**Figure 12:**
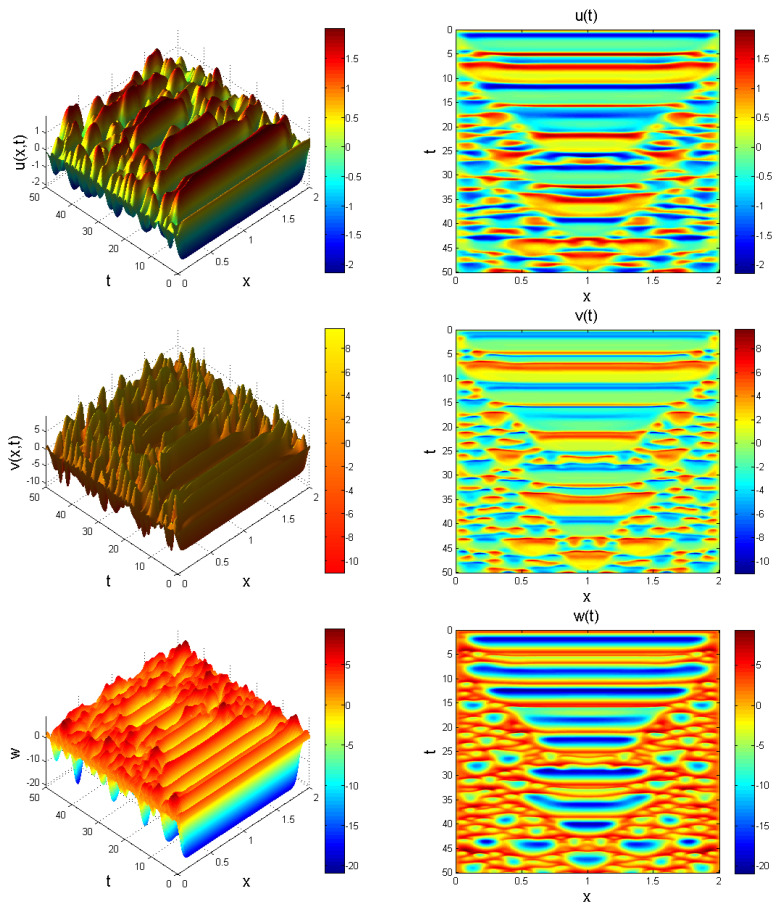
Evolution of system (23) at γ=0.67, x∈[0,2] for t=50. Chaotic distribution is noticeable.

**Figure 13:**
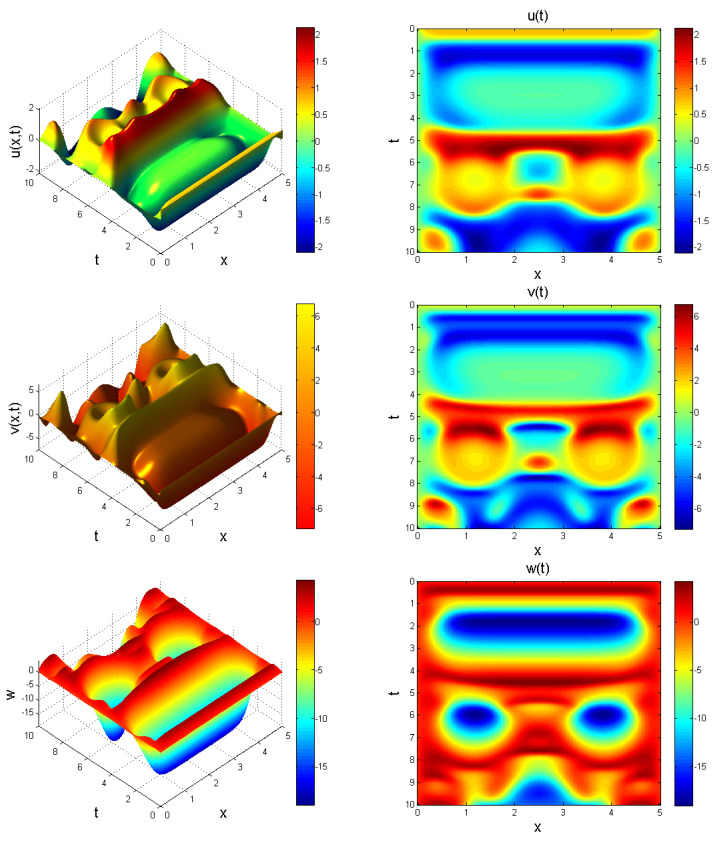
Evolution of system (23) at γ=0.99, x∈[0,5] for t=5. Chaotic behaviour is evident.

In what follows, we provide an extension to system (11) to form a Caputo fractional reaction-diffusion problem as:

(23) $$\begin{array}{ccc} {}^{C}D_{t}^{\gamma }u & = & \Delta^{2}u+b_{1}v-b_{2}u+b_{3}uw,\\ {}^{C}D_{t}^{\gamma }v & = & \Delta^{2}v-b_{4}u\left(t\right)w-b_{5}u+b_{6}vw+b_{7}u,\\ {}^{C}D_{t}^{\gamma }w & = & \Delta^{2}w+b_{8}-b_{9}v^{2}, \end{array}$$

where u=u(x,t), v=v(x,t), w=w(x,t), and $\Delta^{2}=\partial^{2}/\partial x^{2}$ denotes a nonlinear Laplacian operator defined in terms of second order central finite difference operator. We choose x∈[0,L] for *L*, which is enough for waves to propagate. System (23) is solved using the initial function set as:

(24) u0=0.7(ones(N,1)),v0=1(ones(N,1)),w0=0.2(ones(N,1)),

so as to induce a nontrivial result. Behaviour of system (23) showing chaotic evolution is shown in Figure 11, Figure 12 and Figure 13 for different instances of fractional power γ.

## 5. Conclusions

A range of chaotic systems modelled by the Caputo fractional derivatives of order γ are reported in this paper. A Chebyshev spectral method was utilised for the numerical approximation and linear mathematical analysis of the models were also considered. A number of results based on numerical simulations at some instances of fractional index were also reported with amazing chaotic patterns. It was observed that the pattern formation in integer-order systems were similar to the fractional cases.
